# Clinical Efficacy of Temocillin Standard Dosing in Patients Treated with Outpatient Antimicrobial Therapy

**DOI:** 10.3390/pharmaceutics14112289

**Published:** 2022-10-25

**Authors:** Evelyne Van den Broucke, Lore Thijs, Stefanie Desmet, Lotte Vander Elst, Matthias Gijsen, Marnix Mylemans, Otto Van de Gaer, Willy E. Peetermans, Charlotte Quintens, Isabel Spriet

**Affiliations:** 1 Pharmacy Department, University Hospitals Leuven, 3000 Leuven, Belgium; 2 Department of Pharmaceutical and Pharmacological Sciences, KU Leuven, 3000 Leuven, Belgium; 3 Clinical Department of Laboratory Medicine, University Hospitals Leuven, 3000 Leuven, Belgium; 4 Department of Microbiology, Immunology and Transplantation, KU Leuven, 3000 Leuven, Belgium; 5 Department of General Internal Medicine, University Hospitals Leuven, 3000 Leuven, Belgium

**Keywords:** temocillin, dosing, EUCAST, MIC, OPAT at home

## Abstract

In 2020, EUCAST introduced breakpoints for temocillin. Based on these guidelines, reporting of temocillin susceptibility of *Enterobacterales* in the context of complicated urinary tract infections (cUTI) implicates the use of a high dose of temocillin (2 g q8h) constantly. We aimed to evaluate the clinical outcome of patients treated with the standard dose (4 g/day) of temocillin in outpatient parenteral antimicrobial therapy (tOPAT). Demographics, clinical and treatment parameters, and late clinical cure (at day 30 after tOPAT completion) were recorded. Univariate generalised estimating equation analyses, with clinical cure as outcome variable, were performed to evaluate covariate associations. Fifty-seven tOPAT episodes in 50 patients were included with a median antimicrobial treatment duration of 21 (range 10–228) days, and cUTI was the main indication (87.7%). Late clinical cure was achieved in 85.7% of the tOPAT episodes. Non-disseminated infections and minimal inhibitory concentrations (MIC) values ≤ 8 mg/L were associated with good late clinical outcome. In conclusion, a standard temocillin dose (4 g/day) results in good clinical outcomes in the treatment of cUTIs in tOPAT patients. Therefore, our centre concluded that the use of standard temocillin dosing should be continued instead of the high dose for cUTI in non-critically ill patients infected with *Enterobacterales* with an MIC ≤ 4 mg/L.

## 1. Introduction

Temocillin is a small-spectrum beta-lactam antimicrobial, mainly directed against *Enterobacterales*. It shows an outstanding stability against a wide range of beta-lactamases, including extended spectrum beta-lactamases, which is a favourable characteristic in the era of increasing gram-negative resistance [1]. Therefore, it is an attractive treatment alternative for broad-spectrum antimicrobials such as piperacillin-tazobactam, fourth generation cephalosporines and carbapenems in the treatment of infections with beta-lactamase-producing Gram-negative bacilli. As it is highly excreted in urine, it is a preferred treatment option in patients with complicated urinary tract infections (cUTI). Temocillin is frequently used in both clinical and ambulatory settings in Belgium and the United Kingdom, where it has been marketed for many years [1,2,3,4,5,6].

The pharmacokinetic/pharmacodynamic (PK/PD) index related to the efficacy of temocillin, as for other beta-lactam antimicrobials, is the time during which the free drug concentration exceeds the minimum inhibitory concentration (MIC) (%*f*T_>MIC_) [1,7]. The target *%f*T*_>_*_MIC_ for intermittent administration of temocillin has been set at 40% of the dosing interval in previous studies [1,7,8]. There has been some controversy in the past regarding clinical MIC breakpoints and optimal dosing regimens for temocillin [1]. A %*f*T_>MIC_ of 40–50% was found to be associated with bacterial killing and survival in vivo. Monte Carlo simulations performed in critically ill patients showed that 2 g q12h and 2 g q8h regimens both provide a 95% probability of target attainment of 40% *f*T_>MIC_ up to an MIC of 8 mg/L. In the same study, simulations also showed that in less severely ill patients or in specific foci of infection, such as UTI, a daily dose of 4 g should be adequate for strains with an MIC up to 16 mg/L [1].

Recently, the European Committee on Antimicrobial Susceptibility Testing (EUCAST) redefined the categories for susceptibility testing moving from “I” previously defined as “intermediate susceptibility” to “I” now defined as susceptible provided an increased exposure to the antimicrobial agent, i.e., “susceptible, increased exposure” [9]. In 2020, EUCAST introduced the breakpoints and dosing regimens for temocillin; the “S” category has been abandoned when reporting the susceptibility of *Enterobacterales* to temocillin. Strains with an MIC up to 16 mg/L are now categorized as “I” according to the new EUCAST recommendations. Patients infected with these strains should be treated with a high dose, i.e., 2 g q8h. Whereas before the implementation of the new EUCAST guidelines, the standard dose of 2 g q12h was used for strains with an MIC up to 32 mg/L in our hospital [1,10]. The EUCAST breakpoints for temocillin apply to *E. coli*, *Klebsiella* spp. (except *K. aerogenes*) and *P. mirabilis* and is restricted to patients suffering from cUTI, including urosepsis, but excluding patients admitted with severe sepsis and septic shock [11].

In our centre, temocillin is commonly used to treat patients with cUTI, including pyelonephritis, prostatitis and renal cyst infection. Treatment is initiated during hospitalisation, but often completed at home via outpatient parenteral antimicrobial treatment (OPAT). The overall results of our OPAT program were found to be successful, with an excellent clinical cure rate and a low rate of OPAT-related hospital readmissions [12]. In order to assess the updated EUCAST recommendation for temocillin dose augmentation, the purpose of this study was to evaluate the clinical outcome of patients treated with a standard temocillin dose, i.e., 4 g daily, in the outpatient setting.

## 2. Materials and Methods

### 2.1. Design, Setting and Study Participants

A mono-centre, prospective observational study was conducted at the University Hospitals Leuven, a 1950-bed tertiary care centre in Belgium. All OPAT episodes with temocillin (tOPAT) as targeted monotherapy initiated between February 2017 and February 2022 and with complete follow-up registration were included in the study. It was possible that patients received empirical therapy with an alternative broad-spectrum antibiotic prior to tOPAT. Approval for this study was granted by the Ethical Committee of the University Hospitals Leuven (S60847).

### 2.2. tOPAT Program of the University Hospitals Leuven

The OPAT program of the University Hospitals Leuven provides a home-based service in which home care nurses compound and administer parenteral antimicrobials at the patient’s home. The program is based on a multidisciplinary collaboration between the treating physician, the infectious diseases specialist, the microbiologist, the clinical pharmacist and the (home care) nurse, and is coordinated by a clinical pharmacist, who is also referred to as the OPAT coordinator [12].

Temocillin is administered at home through intermittent infusion (2 g q12h, infused over 15 min) or continuously via an elastomeric pump (Baxter Infusor LV10^®^) (4 g q24h, infused over 24 h). In patients with renal insufficiency, the dose is adjusted according to the estimated glomerular filtration rate (eGFR), calculated according to the CKD-EPI formula. Follow-up of the tOPAT patients is provided by completion of a follow-up form by the home care nurse at each visit, which is reviewed weekly by the OPAT coordinator. An end of therapy consultation at the hospital is foreseen to assess clinical outcome and to ensure removal of the venous access device.

### 2.3. Data Collection and Analysis

#### 2.3.1. Demographics, Clinical and Treatment-Related Data

All tOPAT episodes were prospectively registered in a database. Multiple tOPAT episodes of the same patient were analysed independently. Data were extracted from the electronic health record and included demographic data (i.e., age, gender, medical discipline) and clinical and treatment-related data such as dose, administration mode (intermittent or continuous infusion), renal function at start of therapy and at discharge, augmented renal clearance (ARC) at discharge, infection focus, causative pathogen and associated temocillin MIC (Vitek2 (bioMérieux, Marcy-l’Etoile, Lyon, France)), and duration of tOPAT and total antimicrobial treatment. Temocillin MICs were determined by an automated method (Vitek 2). Renal function was expressed as the eGFR,. ARC was defined as an eGFR of more than 96.5 mL/min/1.73 m^2^ [13].

#### 2.3.2. Clinical Outcome

Clinical outcome was independently assessed by two clinical pharmacists (LT and CQ), a microbiologist (SD) and an infectious diseases specialist (WEP). The outcome was rated as either clinical cure or clinical failure. Patients were assessed as clinically cured in case of absence of fever or other signs of infection and if there were no unplanned hospital readmissions for the same clinical problem, as well as no registration of the same infection after completion of tOPAT. Clinical failure was defined as relapse of infection during or after completion of tOPAT. Patients readmitted, whether or not unplanned, due to tOPAT or non-tOPAT-related problems and who nonetheless ended their tOPAT episode were still assessed as either clinically cured or failed according to the abovementioned definition. Patients who presented with tOPAT-related adverse events or complications other than the infection under treatment leading to early discontinuation of tOPAT were registered as non-assessable. The primary outcome was late clinical cure, defined as clinical cure at day 30 after tOPAT completion. Secondary outcomes included clinical cure at the end of treatment and early clinical cure, defined as clinical cure at day 10 after tOPAT completion.

#### 2.3.3. Safety

Also, the tOPAT related adverse event rate was registered, including venous access line related adverse events (LRAE) and adverse drug events (ADE) and analysed as previously described [12]. An adverse event was defined as any untoward medical occurrence in a patient temporally associated, whether causally or not, with the use of tOPAT.

#### 2.3.4. Statistical Analysis

Statistical analyses were performed using IBM SPSS Statistics 27 and R (version 4.0.0, R Core Team, Vienna, Austria). Data were described as count and percentage or median and range as appropriate. Univariate generalised estimating equation (GEE) analyses, with the patient identification number as clustering variable and clinical cure as outcome variable, were performed to evaluate potential associations with (clinically) relevant covariates (age, sex, infection focus, MIC, administration mode, antibiotic duration, OPAT duration, eGFR at start of therapy, eGFR at discharge and ARC at discharge). Separate GEE analyses were performed for late and early clinical cure, which were all set as binomial outcome variables. Statistical significance was assessed based on a *p*-value < 0.05.

## 3. Results

### 3.1. Demographics

Fifty-seven tOPAT episodes in a total of 50 patients were included. Six patients had multiple tOPAT episodes during the five-year study period. The median age was 65 years (range 20–83), and 78% were male. The urology (*n* = 27, 47.4%), general internal medicine (*n* = 15, 26.3%) and nephrology (*n* = 10, 17.5%) wards accounted for the majority of tOPAT discharges. Only one patient was admitted to the intensive care unit during hospital stay. An overview of the demographics is shown in Table 1.

### 3.2. Clinical and Treatment-Related Data

In all OPAT episodes, temocillin was started at the standard dose of 4 g/day. There were no dose adjustments during all the temocillin therapies. Temocillin was administered as an intermittent infusion in the majority of the tOPAT episodes (*n* = 47, 82.5%). The median renal function at OPAT discharge was 75 mL/min/1.73 m^2^ (range 5–140). ARC was present in 10 patients at discharge (median eGFR 118.5 mL/min/1.73 m^2^; range 97–140).

The most common indications for treatment with tOPAT were cUTI (*n* = 50, 87.7%) of which prostatitis (*n* = 21, 36.8%) and urosepsis (*n* = 12, 21.1%) were the most frequently documented subdiagnoses. In the majority of the tOPAT episodes, *E. coli* (*n* = 38, 66.7%) and *K. pneumoniae* (*n* = 10, 17.5%) were the causative pathogens (Table 1). Antimicrobial susceptibility based on the MIC was documented in 46 (80.7%) tOPAT episodes. In six (10.5%) episodes, the causative pathogens were cultured elsewhere (i.e., general practitioner or other hospital); in three (5.3%) episodes, therapy was based on past cultures; in one (1.8%) episode, susceptibility was determined using the disk diffusion method and in one (1.8%) episode, temocillin was started empirically. For most tOPAT episodes, the MIC of the infecting pathogen was ≤4 mg/L (*n* = 32, 69.6%). In four (8.7%), nine (19.6%) and one (2.2%) tOPAT episodes, the MIC of the causative strain was 8, 16 and 32 mg/L, respectively.

The median duration of a tOPAT episode and the median total treatment duration with temocillin were 15 days (range 3–215) and 21 days (range 10–228), respectively. Overall, the 57 tOPAT episodes resulted in 1127 avoided hospitalization days.

### 3.3. Clinical Outcomes

#### 3.3.1. Primary Outcome: Late Clinical Cure

**Figure 1:**
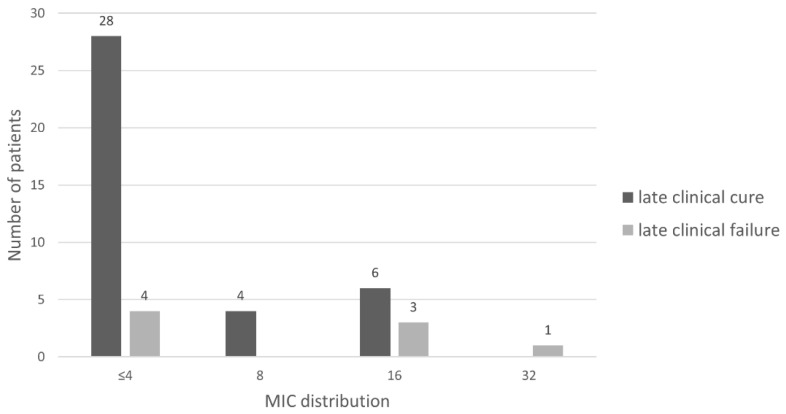
MIC distribution for primary outcome: late clinical cure (*n* = 46). MIC = minimal inhibitory concentration.

Late clinical cure was assessed in 56 of the 57 tOPAT episodes, as the primary outcome was not assessable in one patient. This patient deceased within one month after completion of tOPAT due to non-infectious complications. Late clinical cure was achieved in 85.7% (*n* = 48) of the tOPAT episodes. The distribution of MIC values in relation to late clinical cure is shown in Figure 1. Late clinical failure was registered for 14.3% (*n* = 8) of the tOPAT episodes due to reinfection with the need for re-institution of antimicrobial treatment within 30 days (range: 2–19) after completion of tOPAT. Late clinical failure was ascribed to either inadequate source control (*n* = 6; 75.0%) or too short therapy duration (*n* = 2; 25.0%) as independently assessed by an expert review panel. Patients were considered to have had inadequate source control if there was an inability of definitive control of the source of the ongoing infection process (i.e., no source control procedure was undertaken or procedure was not effective). In the concerning six episodes the probable source contained: an infected cyst in autosomal dominant polycystic kidney disease, Bricker bladder following cystectomy, nephrostomy, collection around transplanted kidney, cholangitis and double J-stent. In seven of the eight episodes with late clinical failure, temocillin was administered intermittently.

Median eGFR at discharge was 77 mL/min/1.73 m^2^ (range 5–140) and 55 mL/min/1.73 m^2^ (range 20–95) for the episodes assessed as clinical cure and clinical failure at day 30 after tOPAT completion, respectively. All patients with ARC (median eGFR 118.5 mL/min/1.73 m^2^; range 97–140) at discharge were assessed as clinically cured at day 30 after tOPAT completion.

Table 2 summarizes the clinically relevant covariates in the total population and in the tOPAT episodes with late clinical cure or late clinical failure, respectively. Additionally, Table 2 reports the associations between the clinical covariates and late clinical cure. Prostatitis, undefined cUTI, epididymitis, pyelonephritis and prostatitis and cystitis infection foci are associated with late clinical cure as compared to urosepsis. Cholangitis is associated with late clinical failure. MIC values ≤ 8 mg/L are significantly associated with late clinical cure (*p* < 0.001). The number of tOPAT episodes with an MIC ≤ 8 mg/L was 32 (84.2%) and 4 (50.0%) for late clinical cure and late clinical failure, respectively. Another significant association was found for tOPAT episodes with ARC at discharge and late clinical cure (*p* < 0.001). None of the other covariates showed significant association with late clinical cure.

#### 3.3.2. Secondary Outcomes: Clinical Cure at the End of Therapy and Early Clinical Cure

The secondary outcomes, clinical cure at the end of treatment and early clinical cure, were achieved in 100% (*n* = 57) and 93.0% (*n* = 53) of the tOPAT episodes, respectively. Therefore, no univariate associations were computed for the clinical cure at the end of treatment. The associations between covariates and early clinical cure are shown in Supplementary Materials Table S1. Early clinical cure was assessed in the total population (i.e., 57 tOPAT episodes). Prostatitis, pyelonephritis, undefined cUTI, epididymitis, pyelonephritis and prostatitis and cystitis infection foci are associated with early clinical cure as compared to patients with urosepsis. Cholangitis is associated with late clinical failure. The median (range) duration of antibiotic therapy was 21 days (10–228) and 28 days (14–42) for early clinical cure and early clinical failure, respectively. Longer antibiotic duration is significantly associated with early clinical failure (*p* < 0.001). Also, ARC at discharge showed a significant association with early clinical cure (*p* < 0.001). None of the other covariates showed significant association with early clinical cure.

### 3.4. Safety

During 6 (10.5%) tOPAT episodes, patients suffered from one or more adverse events. During one (1.8%) tOPAT episode, the patient experienced diarrhoea. During five (8.8%) tOPAT episodes, patients developed LRAEs (i.e., catheter malfunction (*n* = 3), catheter-related infection (*n* = 1) and access site injuries (*n* = 1)). The patient who was diagnosed with a catheter-related infection was readmitted and temocillin therapy was completed in the hospital.

## 4. Discussion

In this series of 57 tOPAT patients, mainly with cUTIs caused by strains with an MIC up to 32 mg/L, a standard temocillin dose of 4 g daily resulted in good clinical outcomes. Good clinical outcomes are demonstrated by a late clinical cure rate of 85.7% and clinical cure rates of 100% at end of therapy (EOT) and 93.0% at day ten after tOPAT completion. Non-disseminated infections, MIC values ≤ 8 mg/L and ARC were associated with good late clinical outcome.

Despite good clinical outcomes, late clinical failure was still observed in eight patients (14.3%). According to an expert review panel, late clinical failure was attributed to either inadequate source control and/or insufficient therapy duration. Although, no association was found between late clinical cure and treatment duration. When the primary outcome is analysed without taking into account the episodes with inadequate source control, a late clinical cure rate of 96.0% (48/50) was achieved.

The univariate GEE analyses demonstrate several associations between covariates and clinical cure. First, prostatitis, undefined cUTI, epididymitis, pyelonephritis and prostatitis and cystitis infection foci seem to be associated with late clinical cure as compared to urosepsis. The same correlation was observed for prostatitis, pyelonephritis, undefined cUTI, epididymitis, pyelonephritis and prostatitis and cystitis foci as compared to urosepsis for early clinical cure. These findings suggest that disseminated (i.e., with bacteraemia) infections result in worse clinical outcome compared to localised infections. Second, cholangitis is associated with both late and early clinical failure. This finding could reflect the fact that temocillin is not first-line therapy for the treatment of abdominal infections. However, these results of associations with infection focus should be interpreted with extreme caution, as they are only based on a small number of patients. Third, for late clinical cure, MIC values ≤ 8 mg/L are significantly associated with clinical cure. Furthermore, no associations were found for eGFR. Interestingly, tOPAT episodes with ARC at discharge were significantly associated with both late and early clinical cure. This finding is not surprising as ARC patients are often younger patients with lesser comorbidities [14]. Longer antibiotic treatment duration was associated with early clinical failure. This result can possibly be explained by the fact that the median of antibiotic treatment days in the early clinical failure group was only assessed on four patients. Whereas the median in early clinical cure group was assessed on 53 patients. Therefore, this result should be interpreted with caution.

For decades, clinical breakpoints and optimal dosing strategies for temocillin have remained debatable. EUCAST has now proposed to use a higher temocillin dose of 2 g q8h for cUTIs caused by *E. coli*, *Klebsiella* spp. (except *K. aerogenes*) and *P. mirabilis* with MICs up to 16 mg/L [11]. This EUCAST dosing recommendation is mainly based on the publications of Laterre et al. and de Jongh et al., who report on the relation between target attainment and clinical cure rate for temocillin treatment [8,15]. Laterre et al. studied pharmacokinetics and target attainment in critically ill patients treated with a daily dose temocillin of 6 g, mainly for ventilator-associated pneumonia or intra-abdominal infection. An overall clinical cure rate of 84% was reported. They concluded, based on a Monte Carlo Simulation, that a dosing regimen of 2 g q8h is sufficient to achieve an average *%f*T*_>_*_MIC_ value of 80% and 40% for an MIC of 16 mg/L and 32 mg/L, respectively. When considering the 95% confidence interval, a *%f*T*_>_*_MIC_ of 50% was found for an MIC of 8 mg/L [15]. De Jongh et al. conducted a similar study with temocillin in a daily dose of 4 g. They observed, also based on a Monte Carlo Simulation, that a dose of 2 g q12h results in a median *%f*T*_>_*_MIC_ of 40% for an MIC of 16 mg/L. Taking into account the 95% confidence interval, they predicted that a dose of 2 g q12h will result in 40% *%f*T*_>_*_MIC_ for an MIC of 8 mg/L in 95% of cases. Clinical cure was achieved for all patients evaluated in the study [8]. In our opinion, extrapolation based on these findings to the setting of cUTI should be carried out carefully. The Monte Carlo Simulations were based on very small sample sizes (*n* = 11 and *n* = 6), all patients were critically ill, and the majority was treated for infections other than UTIs [8,15]. This patient population often faces significant physiological changes, associated with variability in pharmacokinetic parameters, such as an increased volume of distribution related to capillary leakage, hypoalbuminemia, and altered renal function, ranging from ARC to acute kidney injury, all of which may affect antimicrobial exposure [15,16].

Several other more recently conducted studies investigated the clinical efficacy of temocillin in non-critically ill patients. Unfortunately, pharmacokinetic/pharmacodynamic (PK/PD) data are lacking. Clinical cure rates ranging from 79.5–94.0% were reported with a daily dose of 4 g. Although direct comparison with our study is difficult due to differences in definitions and mode of administration, our results are in line with these previously reported clinical cure rates with a daily dose of 4 g [2,3,6,17].

Based on the abovementioned limitations associated with the work of Laterre et al. and de Jongh et al., and reassured by the more recent clinical studies, we questioned the need to increase the current standard dose (4 g daily) to 6 g daily. In our practice, temocillin is used almost exclusively as a targeted treatment of cUTI caused by susceptible *Enterobacterales* in non-critically ill patients, a population in which pharmacokinetic alterations that occur in critically ill patients are not expected. In addition, temocillin is primarily excreted renally with a urinary recovery of 72–82% after 24 h, leading to a high exposure at the site of infection in patients with cUTI [1,2,6]. During a multidisciplinary consensus meeting in our hospital, specific dosing regimens were defined. We decided to treat patients with cUTI caused by *Enterobacterales* with MICs ≤ 4 mg/L with the standard dose of 2 g q12h, as PKPD studies support appropriate target attainment in these patients [2,3,6,8,15,17]. For *Enterobacterales* with MICs of 8 mg/L up to 16 mg/L and for septic patients (i.e., patients presenting with bacteraemia) with a cUTI regardless of the MIC of the causative pathogen, the high dose of 2 g q8h will be applied. These decisions were further supported by the results of our GEE analyses, as MIC values ≤ 8 mg/L and non-disseminated infections, were associated with late clinical cure.

In brief, at our institution, *Enterobacterales* with an MIC ≤ 4 mg/L are reported as being “S”, so that the clinician understands that a standard dose of 2 g q12h is appropriate. Pathogens with an MIC of 8 or 16 mg/L are reported as “I”, indicating that the high dose should be prescribed to ensure appropriate exposure. By doing so, we understand that the wild-type distribution of *E. coli* (with epidemiological cut-off = 16 mg/L) is bisected by the breakpoint, which is against the EUCAST breakpoint setting principles. EUCAST avoids splitting wild type distributions as within the wild type natural and mostly random variation results in uncertainty in MIC testing. By putting the breakpoint at ≤4 mg/L and leaving an I category for MICs of 8 and 16 mg/L we assume that we take into account this analytical uncertainty in MIC testing. As such, exposure remains warranted for bacteria with MICs of 8 and 16 mg/L for temocillin as we will use the high dose in this context. In addition, about 80% of *Enterobacterales* strains isolated at the University Hospitals Leuven showed MICs ≤ 4 mg/L for temocillin. Our more conservative dosing approach (i.e., 4 g/day for MICs ≤ 4 mg/L) is also attractive from a pharmaco-economic point of view, as temocillin is relatively expensive (37 €/2 g, Belgium).

Our study has several limitations that should be taken into account when interpreting the results. First, we included a relatively small sample size. As a result, in combination with the relatively low event rate of clinical failure, associations between covariates and clinical outcome were only tested in a univariate GEE. Second, temocillin was almost exclusively used in the treatment of cUTI, hampering extrapolation of our finding to other types of infections such as intra-abdominal or respiratory infections.

In the future it will be important to follow up on this new dosing protocol prospectively to ensure clinical efficacy, ideally with concurrent pharmacokinetic follow-up, to document the *%f*T*_>_*_MIC_. Some studies have reported pitfalls that may arise with the introduction of the new EUCAST guidelines, such as increased use of broad-spectrum antibiotics [18]. To overcome such undesirable consequences, our centre has implemented several strategies to guide physicians with the use of the new EUCAST guidelines (e.g., new dosing table, electronic prescription schemes, additional comments in the antibiogram, general newsletter, newsflashes for nurses with practical guidelines, follow up of correct use and dosing of antibiotics). It might as well be interesting to compare the exposure and to evaluate the potential clinical benefit of a standard dose continuous infusion regimen (4 g q24h) vs. a high dose intermittent infusion regimen (2 g q8h). Adequate exposure and target attainment might be equally achieved in both dosing protocols since temocillin, as a beta-lactam antimicrobial, is a time-dependent antimicrobial in which PKPD targets are optimized when administered as a continuous infusion [19].

## 5. Conclusions

In conclusion, good clinical outcomes can be achieved with a standard temocillin dose of 4 g per day in non-critically ill patients treated for cUTI, provided adequate source control and duration of therapy. Non-disseminated infections showed an association with clinical cure compared to disseminated infections, and MIC values ≤ 8 mg/L were significantly associated with late clinical cure. Therefore, we propose to retain the standard temocillin dose of 4 g daily for the treatment of non-septic patients with cUTI caused by *Enterobacterales* with an MIC ≤ 4 mg/L; and to implement the high dose of 6 g daily for *Enterobacterales* with MICs of 8 up to 16 mg/L and for septic patients with cUTI regardless of the MIC.

## Figures and Tables

**Table 1:** Summary of demographic, clinical and treatment-related data of all the included tOPAT episodes (*n* = 57).

Category	Subcategory	
Demographics	tOPAT episodes, *n*	57
	Patients, *n*	50
	Patients with 1 tOPAT episode, *n* (%)	44(88.0)
	Patients with 2 tOPAT episodes, *n* (%)	5(10.0)
	Patients with 3 tOPAT episodes, *n* (%)	1(2.0)
	Male/female, *n*	39/11
	Age (years), median (range)	65 (20–83)
Medical discipline	Urology	27 (47.4)
	General internal medicine, *n* (%)	15 (26.3)
	Nephrology, *n* (%)	10 (17.5)
	Cardiology, *n* (%)	2 (3.5)
	Digestive oncology, *n* (%)	1 (1.8)
	Geriatric, *n* (%)	1 (1.8)
	Hepatology, *n* (%)	1 (1.8)
Clinical indication	cUTI, *n* (%)	50 (87.7)
	Prostatitis, *n (%)*	21 (36.8)
	Urosepsis, *n (%)*	12 (21.1)
	Pyelonephritis, *n (%)*	7 (12.3)
	Undefined, *n (%)*	6 (10.5)
	Epididymitis, *n (%)*	2 (3.5)
	Cystitis, *n (%)*	1 (1.8)
	Pyelonephritis + prostatitis, *n (%)*	1 (1.8)
	Cyst infection, *n* (%)	6 (10.5)
	Cholangitis, *n* (%)	1 (1.8)
Causative pathogen(s)	*E. coli*, *n* (%)	38 (66.7)
	*K. pneumonia*, *n* (%)	10 (17.5)
	*K. pneumonia + E. coli*, *n* (%)	2 (3.5)
	*E. cloacae*, *n* (%)	2 (3.5)
	*E. coli + S. marcescens*, *n* (%)	1 (1.8)
	*K. oxytoca*, *n* (%)	1 (1.8)
	*K. oxytoca + E. coli*, *n* (%)	1 (1.8)
	*M. morganii*, *n* (%)	1 (1.8)
	Empirical therapy, *n* (%)	1 (1.8)

tOPAT: temocillin in outpatient parenteral antimicrobial therapy, cUTI: complicated urinary tract infections.

**Table 2:** Demographic, clinical and treatment-related data in the total population and in the tOPAT episodes (*n* = 56) with late clinical cure and late clinical failure, respectively.

	All tOPAT Episodes, *n* = 56	Late Clinical Cure, *n* = 48	Late Clinical Failure, *n* = 8	*p*-Value
Age (years), median (range)	66 (20–83)	63 (20–83)	74 (41–79)	0.17
Female, *n* (%)	13 (23.2)	11 (22.9)	2 (25.0)	0.95
Infection focus				
Prostatitis, *n* (%)	21 (37.5)	21 (43.8)	0 (0)	<0.001
Urosepsis, *n* (%)	12 (21.4)	7 (14.6)	5 (62.5)	reference
Pyelonephritis, *n* (%)	7 (12.5)	6 (12.5)	1 (12.5)	0.065
Undefined cUTI, *n* (%)	6 (10.7)	6 (12.5)	0 (0)	<0.001
Cyst, *n* (%)	5 (8.9)	4 (8.3)	1 (12.5)	0.152
Epididymitis, *n* (%)	2 (3.6)	2 (4.2)	0 (0)	<0.001
Pyelonephritis and prostatitis, *n* (%)	1 (1.8)	1 (2.1)	0 (0)	<0.001
Cystitis, *n* (%)	1 (1.8)	1 (2.1)	0 (0)	<0.001
Cholangitis, *n* (%)	1 (1.8)	0 (0)	1 (12.5)	<0.001
MIC ≤ 8 mg/L, *n* (%)	36 (78.3) ^a^	32 (84.2) ^a^	4 (50.0)	<0.001
Intermittent infusion, *n* (%)	47 (83.9)	40 (83.3)	7 (87.5)	0.92
Duration antibiotic therapy (days), median (range)	21 (10–49)	21 (10–49)	18 (10–42)	0.45
Duration OPAT (days), median (range)	15 (3–43)	15 (3–43)	13 (4–38)	0.86
eGFR at start temocillin (mL/min/1.73 m^2^), median (range)	66 (5–140)	66 (5–140)	51 (20–95)	0.27
eGFR at discharge (mL/min/1.73 m^2^), median (range)	74 (5–140)	76 (5–140)	55 (20–95)	0.46
Augmented renal clearance, *n* (%) ^b^	10 (17.9)	10 (20.8)	0 (0)	<0.001

^a^ MIC values were missing in 10 tOPAT episodes; ^b^ augmented renal clearance is defined as an eGFR_CKD-EPI I_ ≥ 96.5 mL/min/1.73 m^2^. ^a^ *p*-value < 0.05 was considered statistically significant. eGFR: estimated glomerular filtration rate; MIC: minimal inhibitory concentration; tOPAT: temocillin outpatient antimicrobial therapy.

## Data Availability

Data is contained within the article and Supplementary Materials. Additional data is available upon reasonable request.

## Supplementary Materials

The following supporting information can be downloaded at: https://www.mdpi.com/article/10.3390/pharmaceutics14112289/s1, Table S1: Demographic, clinical and treatment-related data in the total population and in the tOPAT episodes with early clinical cure and early clinical failure, respectively.
