# Supplementary material for: Clinical Efficacy of Temocillin Standard Dosing in Patients Treated with Outpatient Antimicrobial Therapy
Source: Pharmaceutics. 2022 Oct 25;14(11):2289. doi: 10.3390/pharmaceutics14112289 (PMC9699557; doi:10.3390/pharmaceutics14112289)
Supplement: Supplementary file 1 [file pharmaceutics-14-02289-s001.zip › pharmaceutics-1972061-supplementary.pdf]

Table S1. Demographic, clinical and treatment-related data in the total population and in the tOPAT episodes (n = 57) with early clinical cure and early clinical failure, respectively.

	All tOPAT episodes, n = 57	Early clinical cure, n = 53	Early clinical failure, n = 4	p-value
Age (years), median (range)	66 (20-83)	66 (20-83)	67 (41-72)	0.92
Female, n (%)	13 (22.8)	12 (22.6)	1 (25.0)	0.76
Infection focus				
Prostatitis, n (%)	21 (36.8)	21 (39.6)	0 (0)	< 0.001
Urosepsis, n (%)	12 (21.)	10 (18.9)	2 (50.0)	reference
Pyelonephritis, n (%)	7 (12.3)	7 (13.2)	0 (0)	< 0.001
Undefined cUTI, n (%)	6 (10.5)	6 (11.3)	0 (0)	< 0.001
Cyst, n (%)	6 (10.5)	5 (9.4)	1 (25.0)	0.33
Epididymitis, n (%)	2 (3.5)	2 (3.8)	0 (0)	<0.001
Pyelonephritis and prostatitis, n (%)	1 (1.8)	1 (1.9)	0 (0)	< 0.001
Cystitis, n (%)	1 (1.8)	1 (1.9)	0 (0)	< 0.001
Cholangitis, n (%)	1 (1.8)	0 (0)	1 (25.0)	< 0.001
MIC ≤ 8 mg/L, n (%)	36 (78.3)[a]	34 (81.0)[a]	2 (50.0)	0.16
Intermittent infusion, n (%)	47 (82.5)	44 (83.0)	3 (75.0)	0.56
Duration antibiotic therapy (days), median (range)	21 (10-228)	21 (10-228)	28 (14-42)	< 0.001
Duration OPAT (days), median (range)	15 (3-215)	15 (3-215)	13 (10-33)	0.14
eGFR at start temocillin (mL/min/1.73 m^2), median (range)	64 (5-140)	67 (5-140)	46 (34-95)	0.18
eGFR at discharge (mL/min/1.73m^2), median (range)	75 (5-140)	76 (5-140)	48 (45-95)	0.10
Augmented renal clearance, n (%)[b]	10 (17.5)	10 (18.9)	0 (0)	< 0.001

[a]MIC values were missing in 11 tOPAT episodes; [b]augmented renal clearance is defined as an $eGFR_{CKD-EPI}$ ≥ 96.5 mL/min/1.73m^2. A p-value < 0.05 was considered statistically significant.
eGFR: estimated glomerular filtration rate; MIC: minimal inhibitory concentration; tOPAT: temocillin outpatient antimicrobial therapy.
